# Radiological control of central venous catheter (CVC) versus electrocardiogram-guided control inserted CVC: confirm with transesophageal echocardiography

**DOI:** 10.1186/cc13314

**Published:** 2014-03-17

**Authors:** F Righetti, G Castellano

**Affiliations:** 1 St Boniface Hospital Verona, Italy

## Introduction

The placement of a central venous catheter (CVC) is now common practice. Control of the seat of the tip occurs through the antero-posterior radiograph of the chest (RX). New techniques have been developed to control the seat of the CVC, to avoid complications during the procedure and the RX control post insertion. The aim of this study is to demonstrate the ECG's P-wave amplitude (PWA) increases as we approach the atrio-caval junction (ACJ) by recording the intracavity and show that this method is more sensitive and specific compared with RX control. To confirm this, we use the direct view of the tip by transesophageal echocardiography (TEE), a method which has the most recognized high sensitivity and specificity [1].

## Methods

In 55 adult patients, hospitalized in the ICU, a CVC was placed. We excluded patients with cardiac arrhythmias or pacemaker wearers. All CVCs were placed with an ultrasound-guided puncture technique of the internal jugular vein (IJV) or subclavian vein (SV). The CVC was introduced by the Seldinger technique. Introducing the CVC along the Seldinger guide links to the same terminal on the cable connection as the intra-cavity derivation ECG (set CVC Certofix B; Braun), in turn connected to the adapter for ECG (Certodyn Universaladapter B; Braun). Then the detection mode of the adapter is converted by the ECG trace outside (through surface electrodes) to the ECG mode intra-cavity and an increase of PWA confirmed the CVC in the vicinity of the ACJ. Through the TEE with esophageal average scan at 120° we measured the distance between the tip and the ACJ. The results were expressed as mean with standard deviation.

## Results

Forty-five CVCs were placed in the IJV while 10 were in the SV. No complications or arrhythmias were detected during the procedure. All CVCs produced an increase in PWA. Where the PWA increased by 25% compared with normal, the TEE scanning tip was 2.5 ± 1.3 cm from the ACJ. Where the PWA increased by 33%, the TEE scanning tip was 1.9 ± 1.1 cm from the ACJ. Where the PWA increased by 50%, the TEE scanning tip was 1.3 ± 0.6 cm from the ACJ. The RX reports have described briefly the presence of the catheter in the superior vena cava without making explicit the exact position relative to the ACJ.

## Conclusion

We have shown the increase in PWA by detecting that the endo-cavity, as we proceed with the insertion of the CVC, corresponds to higher proximity to the ACJ. These results canceled the need for RX control in the future.
